# Supplementary material for: Tidal Flushing Rather Than Non-Point Source Nitrogen Pollution Drives Nutrient Dynamics in A Putatively Eutrophic Estuary
Source: Water (Basel). Author manuscript; Available in PMC 2023 Dec 21. (PMC9926401; doi:10.3390/w15010015)

Supplementary Material to 'Stable isotope assessment of nitrogen pollution underscores the importance of tidal flushing on nutrient dynamics in a New Jersey (USA) estuary'

Johannes R. Krause, Michelle E. Gannon, Autumn J. Oczkowski, Morgan J. Schwartz, Lena K. Champlin, David Steinmann, Martha Maxwell-Doyle, Emily Pirl, Victoria Allen and Elizabeth Burke Watson

Supplementary tables

Table S1. Citations for historic maps and imagery used to develop the qualitative depiction of tidal flushing in Figure 1.

year	Number of inlets	Citation
1797	6	Sotzmann, D. F. (1797). VIII. New Jersey [map].1:500,000, Ebelings Erdbeschreibung und Geschichte von Amerika. Hamburg, Germany: Bey Carl Ernst Bohn. https://mapmaker.rutgers.edu/NJ_1797_Sotzmann.jpg
1812	6	Lewis, S. (1812) New Jersey. Drawn by S. Lewis. Tanner sc. 1:200,000, Boston: Published by Thomas & Andrews. https://mapmaker.rutgers.edu/NJ_1812_Lewis.jpg
1834	6	Finley, A. 1834. New Jersey. In *A New General Atlas, Comprising a Complete Set of Maps, representing the Grand Divisions of the Globe, Together with the several Empires, Kingdoms and States in the World; Compiled from the Best Authorities, and corrected by the Most Recent Discoveries*, Philadelphia.
1860	4	Hopkins, G.M. New York with the vicinities of New York and Philadelphia. 1:158,400. State Geological Survey and US Coast Survey.
1878	3	Colton's New Jersey. 1878. 1:475,200.GW. and C.B. Colton & Co., New York
1889	3	Cook, G.H. 1889. Geological Map of New Jersey from original surveys. 1"=5 miles. Geological Survey of New Jersey.
1906	3	Vermeule, C.C. 1906. Geological Survey of New Jersey. 1"=5 miles.
1918	3	Vermeule, C.C. 1918. Road Map of New Jersey. State of New Jersey, Department of Conservation and Development.1"=4 miles.
1928	4	Cape May to Matasquan River. State of New Jersey, Board of Commerce

		and Navigation, 1:80,000
1940	4	New Jersey, Minor Civil Divisions and Townships. 1940. Bureau of the Census.
1954	3	Land Type Areas of New Jersey. 1954. 1″=4 miles. New Jersey Agricultural Experiment Station, Rutgers University, The State University of New Jersey.
1984	3	Google Earth Pro 7.3.4.8642 (Dec 1984). Barnget Bay-Little Egg Harbor-Great Bay Estuary.
2020	3	Google Earth Pro 7.3.4.8642 (Dec 2020). Barnget Bay-Little Egg Harbor-Great Bay Estuary.

Table S2. Samples collected from Barnegat Bay and analyzed for stable carbon and nitrogen isotopes. *Spartina alterniflora* = SA; *Spartina patens* = SP; *Phragmites australis* = PA; *Zostera marina* = ZM; *Ruppia maritima* = RM; *Ulva* spp. =UL; *Callinectes sapidus* = CS; *Geukensia demissa* = GD; *Ilyanassa obsoleta* = TO; *Fundulus* spp. = FUN. Water column suspended matter (SM) was also analyzed. Sample size (n) is denoted within each column.

Site ID	Latitude	Longitude	Date	Zone	Soil	SA	SP	PA	ZM	RM	UL	CS	GD	TO	FUN	SM	U
1	40.0348	-74.0673	12 Aug 2020	1	5	3	1					5	4		2	1	5
2	39.8641	-74.1394	23 July 2020	2	5	3	3	3				4	4			1	3
3	39.7738	-74.1003	15 July 2020	2	5	1		1			1	4	3	5	4	1	5
4	39.7653	-74.1365	15 July 2020	2	5	2	2	3	3	3	3	X	5	5	5	1	5
5	39.8139	-74.1684	11 Aug 2020	2	5	2		2			3	3	X		5	1	5
6	39.8340	-74.1548	11 Aug 2020	2	5	3						5	2	5	4	1	4
7	39.9714	-74.1187	22 July 2020	1	5	3	3	3				3			5	1	
8	39.5327	-74.3009	7 July 2020	4	5	3	3			3		5			5	1	5
9	39.5461	-74.2775	7 July 2020	4	5	3		3		3	3	X	5	4	5	1	
10	39.6065	-74.3076	8 July 2020	3	5	3						2	5	5	5	1	
11	39.6121	-74.2687	8 July 2020	3	5	1	2	1			2	X	5		5	1	5
12	39.6249	-74.2767	8 July 2020	3	5	3	3	3		1		1	1		5	1	5
13	39.6417	-74.2558	9 July 2020	3	5	3	3	4				1	5	4	5	1	5
14	39.6531	-74.2423	9 July 2020	3	5	3	3			3		2	5		4	1	5
15	39.6789	-74.1969	13 July 2020	2	5	2	3	2				4	5	4	5	1	5
16	39.7075	-74.1877	14 July 2020	2	5	2	2					5	5	5	4	1	5
17	39.7222	-74.1705	14 July 2020	2	5	3						4	3	5	5	1	5
18	39.7286	-74.2108	18 Aug 2020	2	5	3	3	2		3		3	5		4	1	
19	39.7421	-74.1981	14 July 2020	2	5	3	1					3	5	4	4	1	5
20	39.7430	-74.1294	15 July 2020	2	5	3	2		2	2	3	3	4	5	4	1	2
21*	39.5640	-74.4190	3 June 2020	3	5	3	3				3	1	5		5	1	5
22*	39.5153	-74.3295	7 July 2020	4	5	3	3			3	2	2	5		5	1	5
23*	39.4960	-74.4000	11 July 2020	4	5	3	3				3	5	5	5	5	1	5
24*	39.4847	-74.3472	1 July 2020	4	5	2	3				X	X	4	5	5	1	5
25*	39.5217	-74.4306	30 June 2020	4	5	3	3	3				3	5	3	5	1	5
26	39.5497	-74.3498	3 July 2019	4	5	3	3	3		3	3	X	25	25	5	1	5
27	39.5623	-74.2916	3 July 2019	4	5	3	3	3		3	3	X	25	25	5	1	5
28	39.7871	-74.1169	30 July 2019	2	5	3			3		3	X	25	25	5	1	5
29	39.8116	-74.0962	2 July 2019	2	5	3	3	3	3		3	X	25	25	5	1	
30	39.8477	-74.1436	31 July 2019	2	5	3	3	3			3	X	25	25	5	1	5
31	39.8900	-74.1377	17 July 2019	1	5	3	3	3			3	X	25		5	1	5
32	39.9207	-74.1172	16 July 2019	1	5	3	3	3		3	3	X	25		5	1	5
33	39.9898	-74.1162	19 June 2019	1	5	3					3	X			5	1	
34	39.9960	-74.0754	4 July 2019	1	5	3	3	3		3		X			5	1	5
35	40.0580	-74.1250	2 July 2019	1	5	3	3	3				X			5	1	5

Table S3. Citations for images used in Figure 2.

Species	Citation
Spartina alterniflora	Tracey Saxby, Integration and Application Network https://ian.umces.edu/media-library/spartina-spp-salt-marsh-grass/
Spartina patens	Tracey Saxby, Integration and Application Network https://ian.umces.edu/media-library/spartina-patens-saltmeadow-cordgrass/
Phragmites australis	Tracey Saxby, Integration and Application Network https://ian.umces.edu/media-library/phragmites-australis-common-reed-singular/
Ulva spp.	Tracey Saxby, Integration and Application Network https://ian.umces.edu/media-library/ulva-spp-sea-lettuce/
Zostera marina	Diana Kleine, Marine Botany UQ https://ian.umces.edu/media-library/zostera-capricornii/
Ruppia maritima	Jane Thomas, Integration and Application Network https://ian.umces.edu/media-library/ruppia-maritima-vegetative-shoots/
Fundulus spp	Tracey Saxby, Integration and Application Network https://ian.umces.edu/media-library/fundulus-heteroclitus-mummichog-female/
Callinectes sapidus	Jane Thomas, Integration and Application Network https://ian.umces.edu/media-library/callinectes-sapidus-blue-crab-ventral-view-adolescent-female/
Ilyanassa obsoleta	Kim Kraeer, Lucy Van Essen-Fishman https://ian.umces.edu/media-library/littoraria-spp-mangrove-periwinkle/
Geukensia demissa	Dieter Tracey, Department of Water Western Australia https://ian.umces.edu/media-library/mussels-2/
Uca spp.	Kim Kraeer, Lucy Van Essen-Fishman https://ian.umces.edu/media-library/uca-annulipes-mangrove-fiddler-crab/

Table S4. Historic and paired modern samples analyzed for carbon and nitrogen stable isotopes. Museums are abbreviated American Museum of Natural History (AMNH), the Delaware Museum of Natural History (DMNH), the New York Botanical Garden (NYBG), the Cleveland Museum of Natural History (CMNH), and the Academy of Natural Sciences of Philadelphia (ANSP).

Museum	Catalog #	Year	Species	*n*	Location	$\delta^{15}N$ + sd	$\delta^{13}C$ + sd	Zone
NYBG	01816587	1934	*Spartina patens*	2	Bay Head	4.6 ± 0.7	-11.8 ± 0.1	1
NYBG	01816802	1915	*Spartina patens*	2	Point Pleasant	6.7 ± 0.1	-11.0 ± 0.3	1
NYBG	01764718	1895	*Phragmites australis*	2	Bay Head	6.0 ± 0.2	-24.6	1
modern		2020	*Phragmites australis*	5		5.4 ± 1.0	-25.9 ± 0.4	1
modern		2020	*Spartina patens*	6		4.2 ± 0.8	-13.7 ± 0.4	1
NYBG	01786908	1950	*Spartina alterniflora*	3	Forked River	3.9 ± 0.2	-10.5 ± 0.1	2
NYBG	01764717	1946	*Phragmites australis*	2	Harvey Cedars	3.1 ± 0.2	-23.6 ± 0.1	2
NYBG	01764753	1937	*Phragmites australis*	2	Waretown	6.2 ± 0.1	-25.4 ± 0.2	2
AMNH	100680	1962	*Modiolus demissus*	1	Barnegat Light	14.8		2
AMNH	100692	1962	*Ilyanassa obsoleta*	5	Harvey Cedars	8.6 ± 0.9		2
AMNH	172622	1963	*Spisulua solidissima*	1	Barnegat Light	10.9		2
AMNH	293587	1965	*Spisulua solidissima*	1	Lavalette	17.2		2
modern		2020	*Spartina patens*	9	Barnegat Inlet	5.6 ± 3.4	-14.3 ± 1.1	2
modern		2020	*Phragmites australis*	9	Barnegat Inlet	7.7 ± 2.2	-25.4 ± 0.6	2
modern		2020	*Spartina alterniflora*	14	Barnegat Inlet	5.8 ± 2.3	-13.5 ± 1.1	2
modern		2020	*Geukensia demissa*	13	Barnegat Inlet	7.6 ± 1.0	-20.9 ± 1.9	2
modern		2020	*Ilyanassa obsoleta*	11	Barnegat Inlet	7.9 ± 1.1	-14.8 ± 1.1	2
CMNH	241229	1940	*Spartina patens*	2	Beach Haven	4.8 ± 0.4	-11.5 ± 0.2	3
DMNH	67639	1943	*Ilyanassa obsoleta*	2	Beach Haven	13.8		3
DMNH	37748	1957	*Mytilus edulis*	2	Beach Haven	14.0 ± 1.3		3
DMNH	102767	1961	*Crassostrea virginica*	1	Beach Haven	13.3		3
DMNH	38345	1970	*Mya arenaria*	1	Beach Haven	12.4		3
DMNH	110611	1975	*Ilyanassa obsoleta*	4	Beach Haven	12.9 ± 0.5		3
modern		2020	*Spartina patens*	5	Little Egg Harbor	2.7 ± 0.8	-14.1 ± 0.2	3
modern		2020	*Ilyanassa obsoleta*	3	Little Egg Harbor	7.1 ± 0.4	-15.2 ± 1.3	3
ANSP	16491	1880	*Ilyanassa obsoleta*	2	Egg Harbor	11.9 ± 1.4		3
ANSP	159286	1932	*Ilyanassa obsoleta*	1	Egg Harbor	14.7		4
ANSP	262992	1954	*Ilyanassa obsoleta*	1	Egg Harbor	13.8		4
modern		2020	*Ilyanassa obsoleta*	5	Great Bay	9.1 ± 1.5	-14.8 ± 1.5	4
NYBG	01816713	1890	*Spartina patens*	2		2.0 ± 1.3	-11.0	4

Table S5: Results of tests for difference of mean δ^{15}N and δ^{13}C between regions. P-values are reported for ANOVA, Kruskal-Wallis, Tukey's and Dunn's test results (significant results in bold). Effect sizes are reported as F-statistics (ANOVA) or eta^2 (Kruskal-Wallis).

					Post-hoc test for differences between regions					
	Variable	Test	*p*	Effect size	1 vs 2	1 vs 3	1 vs 4	2 vs 3	2 vs 4	3 vs 4
δ^{15}N	SPM	ANOVA	**0.008**	4.68	0.12	0.97	**0.05**	**0.05**	0.92	**0.03**
	Soil	Kruskal-Wallis	0.19	0.06						
	S. alterniflora	ANOVA	**0.03**	3.26	**0.03**	0.87	0.21	0.26	0.91	0.67
	S. patens	Kruskal-Wallis	0.1	0.11						
	P. australis	ANOVA	**0.04**	3.36	0.61	0.98	**0.03**	0.89	0.16	0.09
	Ulva	ANOVA	0.51	0.71						
	C. sapidus	ANOVA	**0.003**	5.76	0.08	0.46	0.71	**0.003**	0.65	0.09
	Fundulus	Kruskal-Wallis	**0.02**	0.2	**0.03**	1	0.25	0.09	1	0.46
	G. demissa	ANOVA	**0.04**	3.13	0.72	0.96	0.29	0.19	0.61	0.04
	I. obsoleta	ANOVA	0.05	3.02						
	Uca	ANOVA	0.21	1.23						
δ^{13}C	SPM	ANOVA	**0.001**	7.34	0.22	**0.02**	**0.007**	0.35	**0.02**	0.77
	Soil	Kruskal-Wallis	**0.006**	0.31	1.0	0.07	**0.01**	0.55	0.12	1.0
	S. alterniflora	Kruskal-Wallis	0.64	0.56						
	S. patens	Kruskal-Wallis	0.28	0.03						
	P. australis	ANOVA	0.33	1.25						
	Ulva	ANOVA	0.32	1.3						
	C. sapidus	Kruskal-Wallis	0.33	0.01						
	Fundulus	ANOVA	**0.03**	3.49	0.28	0.21	**0.02**	0.71	0.15	0.83
	G. demissa	ANOVA	**0.05**	2.96	0.59	**0.03**	0.23	0.29	0.98	0.57

	Variable	Test	*p*	Effect size	Post-hoc test for differences between regions					
					1 vs 2	1 vs 3	1 vs 4	2 vs 3	2 vs 4	3 vs 4
$\delta^{15}N$	SPM	ANOVA	**0.008**	4.68	0.12	0.97	**0.05**	**0.05**	0.92	**0.03**
	Soil	Kruskal-Wallis	0.19	0.06						
	S. alterniflora	ANOVA	**0.03**	3.26	**0.03**	0.87	0.21	0.26	0.91	0.67
	S. patens	Kruskal-Wallis	0.1	0.11						
	P. australis	ANOVA	**0.04**	3.36	0.61	0.98	**0.03**	0.89	0.16	0.09
	Ulva	ANOVA	0.51	0.71						
	C. sapidus	ANOVA	**0.003**	5.76	0.08	0.46	0.71	**0.003**	0.65	0.09
	Fundulus	Kruskal-Wallis	**0.02**	0.2	**0.03**	1	0.25	0.09	1	0.46
	G. demissa	ANOVA	**0.04**	3.13	0.72	0.96	0.29	0.19	0.61	0.04
	I. obsoleta	ANOVA	0.05	3.02						
	Uca	ANOVA	0.21	1.23						
	I. obsoleta	ANOVA	0.89	0.11						
	Uca	ANOVA	0.18	1.87						

Table S6. Salt marsh soil core profiles, annotated by horizon. Total C, N, and %OC were determined and C:N ratios (by weight and molar) as well as carbon stocks were computed.

Site	Horizon	Top cm	Bot cm	Total C	Total N	OC %	C/N	C/N molar	OD Db, g/cc	carbon Pool, kg/m²	carbon 100cm	carbon 200cm
Bestpitch	Oe	0	17	16.68	0.78	16.7	21	25	0.25	7.10	7.10	7.10
	Oi	17	26	20.37	0.86	20.4	24	28	0.19	3.49	3.49	3.49
	Oese1	26	54	11.56	0.69	11.6	17	20	0.61	19.81	19.81	19.81
	Oese2	54	90	6.78	0.43	6.8	16	18	0.71	17.38	17.38	17.38
	Cseg1	90	117	4.48	0.32	4.5	14	16	1.07	13.00	4.82	13.00
	Cseg2	117	149	6.61	0.58	6.6	11	13	0.7	14.78		14.78
	Cseg3	149	217	3.46	0.28	3.5	12	14	0.72	17.14		12.85
Bestpitch	Oi	0	10	14.42	0.63	14.4	23	27	0.36	5.18	5.18	5.18
	Oise	10	24	14.13	0.63	14.1	22	26	0.41	8.09	8.09	8.09
	Oese1	24	33	6.47	0.4	6.5	16	19	0.63	3.69	3.69	3.69
	Oese2	33	41	7.27	0.46	7.3	16	18	0.66	3.85	3.85	3.85
	Oese3	41	60	7.55	0.51	7.6	15	17	0.69	9.96	9.96	9.96
	Oese4	60	87	6.42	0.34	6.4	19	22	0.78	13.48	13.48	13.48
	Oese5	87	156	7.52	0.39	7.5	19	22	0.69	35.71	6.73	35.71
	Cseg1	156	183	6.02	0.37	6	16	19	0.7	11.34		11.34
Pawcatuck	Oe	0	17	20.04	1.31	20	15	18	0.36	12.24	12.24	12.24
	Oese1	17	33	10.7	0.71	10.7	15	18	0.65	11.13	11.13	11.13
	Oese2	33	52	10.47	0.59	10.5	18	21	0.5	9.98	9.98	9.98
	Oese3	52	70	7.22	0.41	7.2	18	21	0.63	8.16	8.16	8.16
	Oese4	70	89	7.67	0.4	7.7	19	22	0.67	9.80	9.80	9.80
	2Cseg	89	130	1.05	0.03	1.1	35	41	1.65	7.44	2.00	7.44
Transquaking	Oa	0	8	18.72	1.46	18	13	15	0.31	4.46	4.46	4.46
	Oe	8	19	29.89	1.59	29.9	19	22	0.03	0.99	0.99	0.99
	Oese	19	34	18.52	1.03	18.5	18	21	0.6	16.65	16.65	16.65
	Cseg	34	50	13.47	0.67	13.5	20	23	0.68	14.69	14.69	14.69
	O'ese1	50	113	18.53	0.99	18.5	19	22	0.57	66.43	52.73	66.43
	O'ese2	113	134	17.04	0.7	17	24	28	0.57	20.35		20.35
	C'seg	134	160	12.81	0.57	12.8	22	26	0.68	22.63		22.63
	2Oaseb	160	176	6.97	0.25	7	28	33	0.6	6.72		6.72
	2Aseb	176	182	1.34	0.04	1.3	34	39	0.88	0.69		0.69
Boxiron tax.	Oise	0	13	16.07	1.04	16.1	15	18	0.26	5.44	5.44	5.44
	Oese1	13	23	11.93	0.79	11.9	15	18	0.48	5.71	5.71	5.71
	Cg	23	40	10.79	0.71	10.8	15	18	0.19	3.49	3.49	3.49
	Cseg1	40	69	7.45	0.54	7.4	14	16	0.45	9.66	9.66	9.66
	Cseg2	69	86	4.75	0.41	4.8	12	14	0.66	5.39	5.39	5.39
	Cseg3	86	116	11.12	0.58	11.1	19	22	0.32	10.66	4.97	10.66
	Cseg4	116	132	10.61	0.55	10.6	19	22	0.29	4.92		4.92
	O'ese1	132	158	12.95	0.68	12.9	19	22	0.31	10.40		10.40
	O'ese2	158	178	20.35	1.12	20.4	18	21	0.25	10.20		10.20
	Oase1	178	198	23.23	1.27	23.2	18	21	0.21	9.74		9.74
	Oase2	198	211	16.26	0.85	16.3	19	22	0.22	4.66		0.72

	C'seg1	211	232	10.66	0.56	10.6	19	22	0.34	7.57		
	C'seg2	232	247	6.77	0.53	6.7	13	15	0.42	4.22		
Bestpitch	Oi1	0	10	26.31	1.36	26.3	19	23	0.13	3.42	3.42	3.42
	Oi2	10	20	24.9	1.21	24.9	21	24	0.12	2.99	2.99	2.99
	Oese	20	31	13.64	0.76	13.6	18	21	0.22	3.29	3.29	3.29
	Cseg1	31	44	8.27	0.52	8.3	16	19	0.26	2.81	2.81	2.81
	Cseg2	44	97	7.49	0.43	7.5	17	20	0.55	21.86	21.86	21.86
	O'ese1	97	113	11.86	0.63	11.9	19	22	0.34	6.47	1.21	6.47
	O'ese2	113	156	11.78	0.6	11.8	20	23	0.28	14.21		14.21
	2Cseg1	156	176	9.24	0.55	9.2	17	20	0.4	7.36		7.36
	2Cseg2	176	197	7.08	0.48	7.1	15	17	0.46	6.86		6.86
	2Cseg3	197	212	8.35	0.55	8.4	15	18	0.35	4.41		0.88
	2Cseg4	212	226	5.79	0.41	5.8	14	16	0.44	3.57		
Broadkill	Oise	0	17	21.7	1.11	21.7	20	23	0.19	7.01	7.01	7.01
	Cg	17	29	8.26	0.45	8.3	18	21	0.31	3.09	3.09	3.09
	Cseg1	29	44	1.34	0.11	1.3	12	14	1.38	2.69	2.69	2.69
	Cseg2	44	55	11.21	0.68	11.2	16	19	0.46	5.67	5.67	5.67
	Cseg3	55	66	8.97	0.51	9	18	21	0.35	3.47	3.47	3.47
	Cseg4	66	76	6.24	0.48	6.2	13	15	0.49	3.04	3.04	3.04
	Cseg5	76	106	7.17	0.49	7.2	15	17	0.49	10.58	8.47	10.58
	Cseg6	106	135	8.35	0.51	8.4	16	19	0.42	10.23		10.23
	Cseg7	135	160	8.38	0.55	8.4	15	18	0.36	7.56		7.56
	Cseg8	160	176	7.92	0.46	7.9	17	20	0.48	6.07		6.07
	Oase	176	209	12.67	0.64	12.7	20	23	0.3	12.57		9.14
	C'seg	209	220	8.57	0.54	8.6	16	19	0.48	4.54		
Appoquinimink tax.	Aseg	0	17	4.75	0.28	4.8	17	20	0.83	6.77	6.77	6.77
	Cseg1	17	25	5.24	0.31	5.2	17	20	0.57	2.37	2.37	2.37
	Cseg2	25	41	6.3	0.35	6.3	18	21	0.4	4.03	4.03	4.03
	Acseg	41	70	3.56	0.22	3.6	16	19	0.79	8.25	8.25	8.25
	Cseg3	70	96	4.66	0.28	4.7	17	19	0.49	5.99	5.99	5.99
	Oese	96	126	8.46	0.39	8.5	22	25	0.57	14.54	1.94	14.54
	Oase1	126	142	14	0.63	14	22	26	0.32	7.17		7.17
	Oase2	142	183	13.84	0.7	13.8	20	23	0.34	19.24		19.24
	Oase3	183	213	10.95	0.62	11	18	21	0.29	9.57		5.42
	Oase4	213	228	13.81	0.75	13.8	18	21	0.31	6.42		
Boxiron tax.	Oise	0	24	10.09	0.55	10.1	18	21	0.4	9.70	9.70	9.70
	Cseg1	24	41	7.92	0.44	7.9	18	21	0.33	4.43	4.43	4.43
	Cseg2	41	68	7.43	0.39	7.4	19	22	0.34	6.79	6.79	6.79
	Cseg3	68	80	4.15	0.26	4.2	16	19	0.72	3.63	3.63	3.63
	Cseg4	80	109	2.85	0.16	2.9	18	21	0.59	4.96	3.42	4.96
	2Cseg5	109	148	0.11	0.01	0.1	11	13	1.45	0.57		0.57
	2Cseg6	148	208	0.01		0.01			1.45	0.09		0.08
	2Cseg7	208	223	0.04	0.02	0.01	2	2	1.48	0.02		
Boxiron tax.	Oise	0	13	13.87	0.77	13.9	18	21	0.26	4.70	4.70	4.70
	Oese	13	21	10.74	0.56	10.7	19	22	0.28	2.40	2.40	2.40
	Cseg1	21	38	9.73	0.54	9.7	18	21	0.28	4.62	4.62	4.62

	Cseg2	38	60	8.47	0.45	8.5	19	22	0.31	5.80	5.80	5.80
	Cseg3	60	95	7.93	0.54	7.9	15	17	0.38	10.51	10.51	10.51
	Cseg4	95	133	5.53	0.38	5.5	15	17	0.48	10.03	1.32	10.03
	Cseg5	133	200	2.25	0.17	2.3	13	15	0.73	11.25		11.25
	ACsegb	200	232	4.03	0.29	4	14	16	0.59	7.55		
Bestpitch	Oise	0	15	17.62	0.91	17.6	19	23	0.19	5.02	5.02	5.02
	Oese1	15	34	14.8	0.69	14.8	21	25	0.19	5.34	5.34	5.34
	Oese2	34	74	14.5	0.75	14.5	19	23	0.32	18.56	18.56	18.56
	Oase	74	103	11.33	0.54	11.3	21	24	0.23	7.54	6.76	7.54
	Cseg1	103	149	6.25	0.35	6.3	18	21	0.49	14.20		14.20
	Cseg2	149	187	7.98	0.4	8	20	23	0.28	8.51		8.51
	Cseg3	187	224	6.39	0.35	6.4	18	21	0.73	17.29		6.07
Bestpitch	Oi	0	15	13.61	0.83	13.6	16	19	0.23	4.69	4.69	4.69
	Oe	15	36	17.81	0.99	17.8	18	21	0.19	7.10	7.10	7.10
	Oese	36	53	8.62	0.47	8.6	18	21	0.3	4.39	4.39	4.39
	Cseg1	53	81	5.58	0.36	5.6	16	18	0.38	5.96	5.96	5.96
	Cseg2	81	94	4.96	0.31	5	16	19	0.5	3.25	3.25	3.25
	Cseg3	94	117	3.08	0.22	3.1	14	16	0.6	4.28	1.12	4.28
	Cseg4	117	190	1.78	0.15	1.8	12	14	0.86	11.30		12.85
	Cseg5	190	218	1.84	0.13	1.8	14	17	0.86	4.33		1.55
Bestpitch	Oise	0	18	15.89	0.91	15.9	17	20	0.25	7.16	7.16	7.16
	Oese	18	31	15.69	0.74	15.7	21	25	0.19	3.88	3.88	3.88
	Oase	31	63	11.58	0.56	11.6	21	24	0.29	10.76	10.76	10.76
	Cseg1	63	84	6.48	0.37	6.5	18	20	0.32	4.37	4.37	4.37
	Cseg2	84	108	3.34	0.23	3.3	15	17	0.64	5.07	3.38	5.07
	Cseg3	108	124	4.88	0.3	4.9	16	19	0.31	2.43		2.43
	Cseg4	124	175	1.97	0.15	2	13	15	0.81	8.26		12.31
	2Cg	175	219	0.26	0.02	0.3	13	15	1.27	1.68		0.95
Boxiron tax.	Oise	0	26	11.78	0.73	11.8	16	19	0.3	9.20	9.20	9.20
	Cseg1	26	45	8.48	0.56	8.5	15	18	0.3	4.85	4.85	4.85
	Cseg2	45	87	6.22	0.48	6.2	13	15	0.39	10.16	10.16	10.16
	Cseg3	87	101	8.14	0.49	8.1	17	19	0.37	4.20	3.90	4.20
	Cseg4	101	120	8.94	0.49	8.9	18	21	0.3	5.07		5.07
	Cseg5	120	142	8.51	0.53	8.5	16	19	0.33	6.17		6.17
	Cseg6	142	165	3.7	0.31	3.7	12	14	0.3	2.55		2.55
	Cg	165	210	2.42	0.24	2.4	10	12	0.3	3.24		2.52
Boxiron tax.	Oise	0	24	14.82	0.91	14.8	16	19	0.28	9.95	9.95	9.95
	Cseg1	24	48	7.78	0.51	7.8	15	18	0.4	7.49	7.49	7.49
	Cseg2	48	61	10.95	0.77	11	14	17	0.27	3.86	3.86	3.86
	Cseg3	61	77	11.1	0.85	11.1	13	15	0.36	6.39	6.39	6.39
	Cseg4	77	96	10.82	0.66	10.8	16	19	0.32	6.57	6.57	6.57
	Oase	96	106	16.7	0.91	16.7	18	21	0.26	4.34	1.74	4.34
	2Aseb	106	123	3.23	0.15	3.2	22	25	0.85	4.62		4.62
	2Eb	123	139	0.39	0.02	0.4	20	23	1.66	1.06		1.06
	2Bhsb	139	176	0.49	0.01	0.5	49	57	1.7	3.15		3.15
Boxiron tax.	Oise	0	20	14.47	0.83	14.5	17	20	0.22	6.38	6.38	6.38
	Cseg1	20	46	9.39	0.62	9.4	15	18	0.29	7.09	7.09	7.09

	Cseg2	46	61	9.87	0.63	9.9	16	18	0.27	4.01	4.01	4.01
	Cseg3	61	105	5.33	0.51	5.3	10	12	0.4	9.33	8.27	9.33
	Cseg4	105	119	6.05	0.51	6.1	12	14	0.35	2.99		2.99
	Cseg5	119	156	3.97	0.36	4	11	13	0.54	7.99		7.99
	Cseg6	156	184	8.72	0.56	8.7	16	18	0.4	9.74		9.74
	Cseg7	184	218	6.92	0.5	6.9	14	16	0.4	9.38		4.42
Transquaking	Oise	0	18	23.7	1.18	23.7	20	23	0.15	6.40	6.40	6.40
	Oese	18	43	14.22	0.86	14.2	17	19	0.22	7.81	7.81	7.81
	Cseg	43	69	7.58	0.48	7.6	16	18	0.37	7.31	7.31	7.31
	O'se1	69	110	12.84	0.75	12.8	17	20	0.28	14.69	11.11	14.69
	O'se2	110	120	15.43	0.94	15.4	16	19	0.3	4.62		4.62
	Oase	120	130	13.02	0.44	13	30	35	0.6	7.80		7.80
	2Asegb	130	152	2.18	0.07	2.2	31	36	6.78	6.78		6.78
	2Bhseb	152	182	1.03	0.03	1	34	40	4.20	4.20		4.20
Saltpond	Aseg	0	10	7.16	0.41	7.2	17	20	0.44	3.17	3.17	3.17
	Cseg1	10	21	0.91	0.03	0.9	30	35	0.72	0.71	0.71	0.71
	Cseg2	21	59	0.1	0.01	0.1	10	12	1.35	0.51	0.51	0.51
	Acseg	59	67	0.05	0.03	0.1	2	2	1.45	0.12	0.12	0.12
	C'seg	67	87	0.07	0.01	0.1	7	8	1.48	0.30	0.30	0.30
	AC'seg	87	99	0.07	0.01	0.1	7	8	1.43	0.17	0.17	0.17
	C''seg	99	160	0.02	0.001	0.02	20	23	1.47	0.18	0.00	0.18
Mispillion tax.	Oise	0	18	23.99	1.15	24	21	24	0.11	4.75	4.75	4.75
	Oese1	18	37	17.28	0.88	17.3	20	23	0.18	5.92	5.92	5.92
	Oese2	37	54	14.87	0.83	15	18	21	0.17	4.34	4.34	4.34
	Oase1	54	76	24.1	1.17	24.1	21	24	0.12	6.36	6.36	6.36
	Oase2	76	116	14.16	0.74	14.2	19	22	0.2	11.36	6.82	11.36
	2Agb	116	127	3.47	0.12	3.5	29	34	0.88	3.39		3.39
	2Egb	127	134	1.97	0.13	2	15	18	1.28	1.79		1.79
	2Bhsb	134	147	2.52	0.15	2.5	17	20	1.01	3.28		3.28
	2BCgb	147	190	0.33	0.01	0.3	33	38	1.8	2.32		2.32

Supplementary Figures

Figure S1. Interpolations of water quality parameters measured in Barnegat Bay by NJ DEP for 2014-2021. The number of measurements collected are shown next to the sites. Imagery source: Esri World Imagery acquired in June 2020.

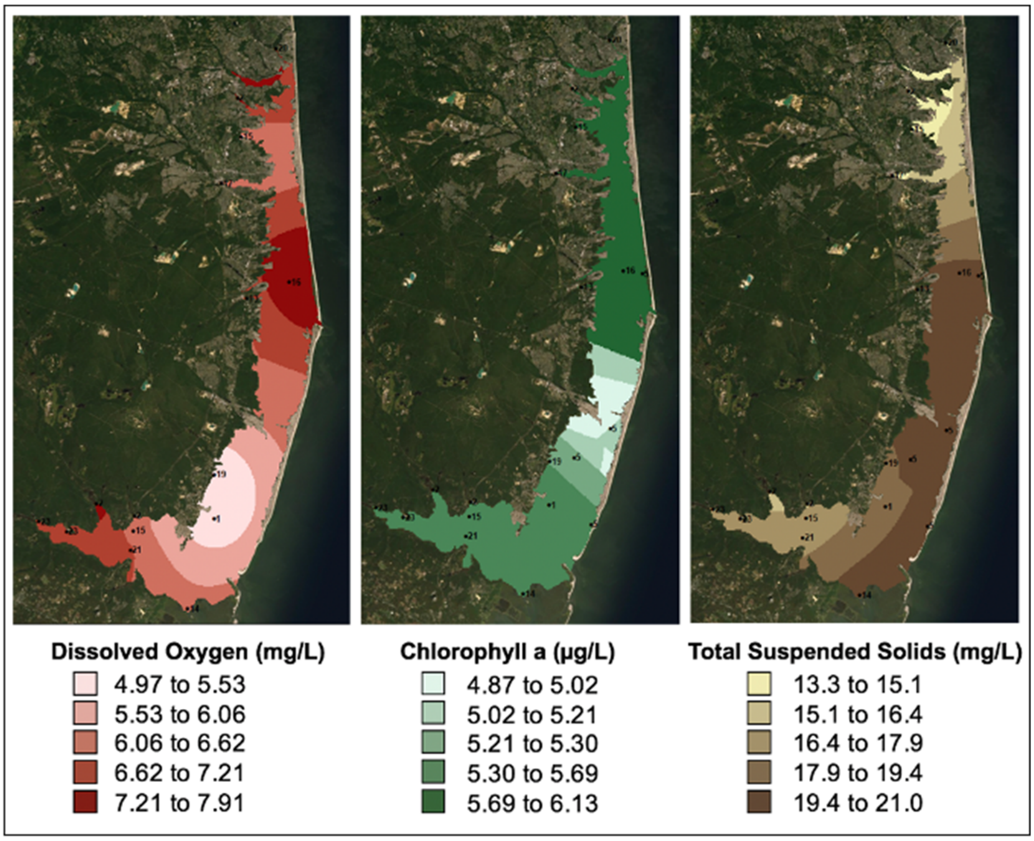

Figure S2. Suspended particulate matter N and C isotopes. Global Moran's I test in ArcGIS: SPM N: z-score = 1.45, *p*-value = 0.146; SPM C: z-score = 0.016, *p*-value = 0.987. Imagery source: Esri World Imagery acquired in June 2020.

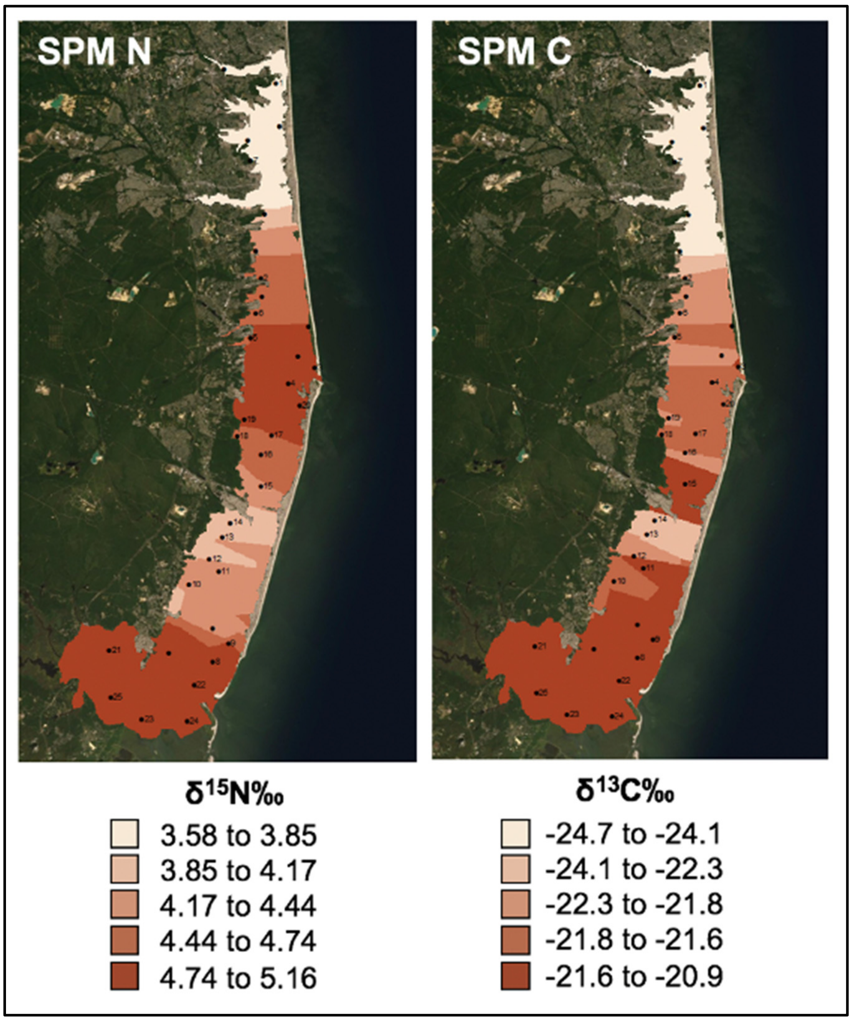

Figure S3. Change of the C/N ratio over core depth, representing changes of the relative C to N inputs over time. The map of C/N slope is the coefficient of a linear regression of the C/N ratio with respect to depth, and red indicates an increased ratio deeper in the core while blue indicates a decreased ratio downcore. Imagery source: Esri World Imagery acquired in June 2020.

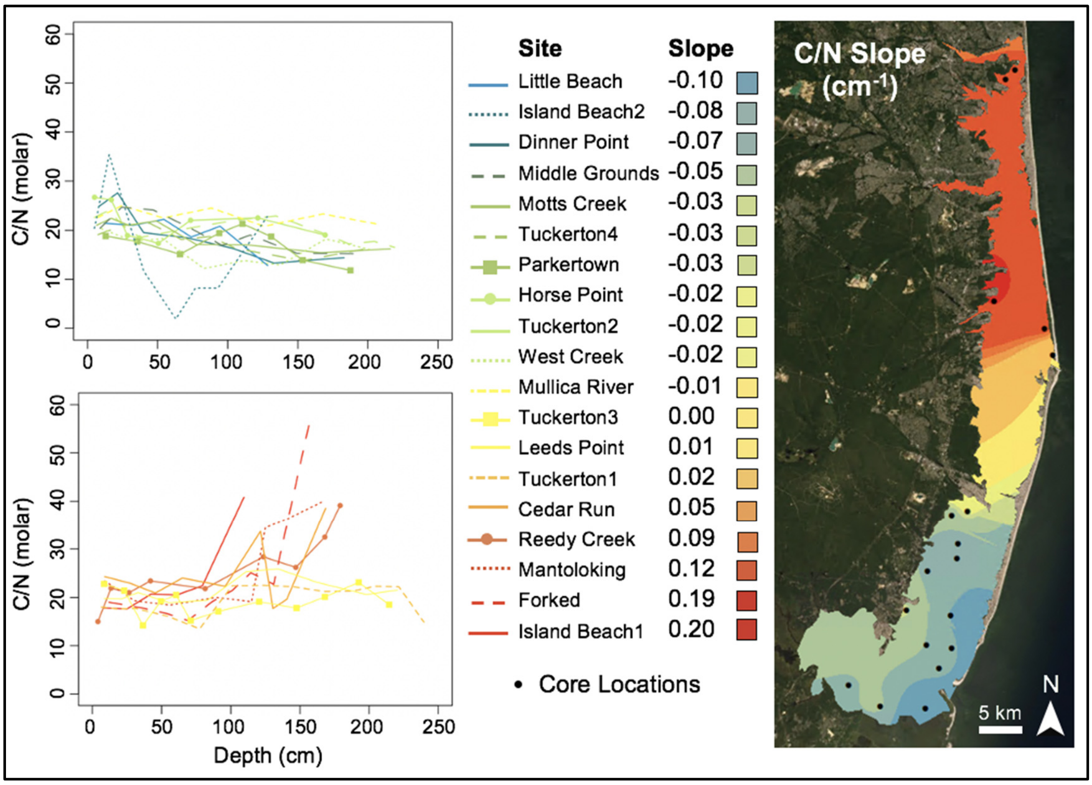

Figure S4. Measurements of total soil carbon and carbon to nitrogen ratio in the surface of marsh sediment cores (corresponding to 0-100cm depth). Imagery source: Esri World Imagery acquired in June 2020.

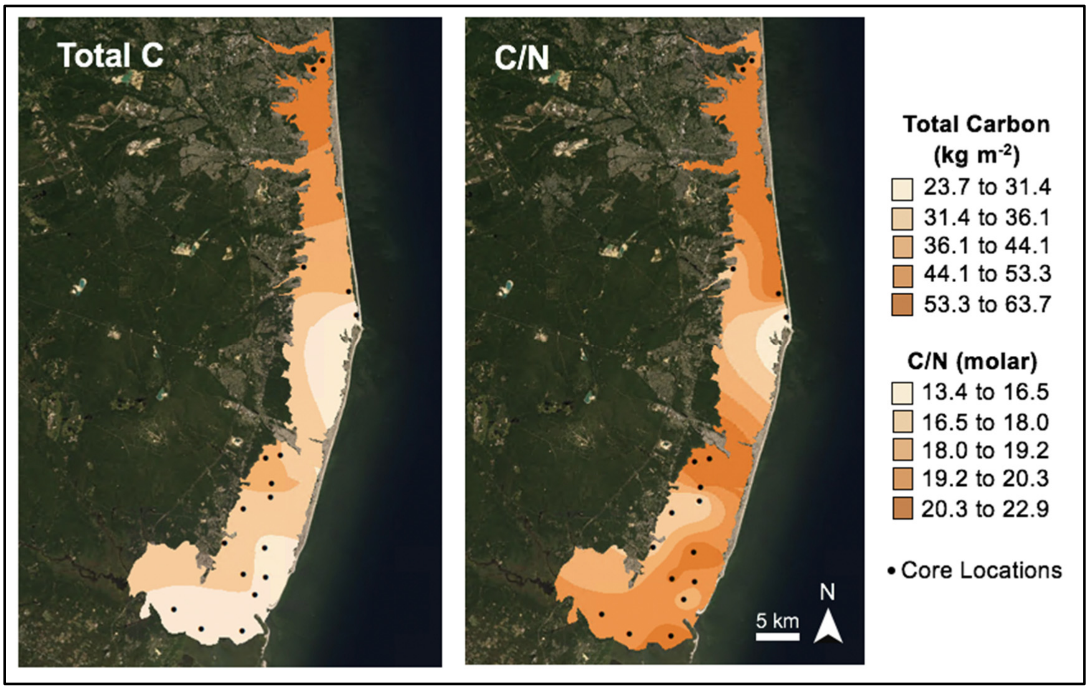

Supplement: Supplement1 [file NIHMS1869035-supplement-Supplement1.zip › water-2070620-supplementary.pdf]
